# Methylmercury exposure, genetic variation in metabolic enzymes, and the risk of glioma

**DOI:** 10.1038/s41598-019-47284-4

**Published:** 2019-07-26

**Authors:** Jordan H. Creed, Noah C. Peeri, Gabriella M. Anic, Reid C. Thompson, Jeffrey J. Olson, Renato V. LaRocca, Sajeel A. Chowdhary, John D. Brockman, Travis A. Gerke, Louis B. Nabors, Kathleen M. Egan

**Affiliations:** 1 Department of Cancer Epidemiology, H. Lee Moffitt Cancer Center & Research Institute, Inc., Tampa, FL 33612 USA; 2 Department of Neurological Surgery, Vanderbilt University Medical Center, Nashville, TN 37232 USA; 3 Department of Neurosurgery, Emory School of Medicine, Atlanta, GA 30322 USA; 4 Norton Cancer Institute, Louisville, KY 40202 USA; 5 Neuro-Oncology Program, Lynn Cancer Institute, 701 NW 13th Street, Boca Raton, FL 33486 USA; 6 University of Missouri Research Reactor, University of Missouri, Columbia, MO 65211 USA; 7 Neuro-oncology Program, University of Alabama at Birmingham, Birmingham, AL 35294 USA

**Keywords:** Cancer epidemiology, CNS cancer

## Abstract

Methylmercury (MeHg) is an environmental neurotoxin with human exposure mainly from dietary intake of contaminated fish. Exposure to MeHg has been implicated in neurological damage, but research on its role in cancers, specifically glioma, is limited. In a glioma case-control study, we examined associations between toenail mercury (Hg) and glioma risk. We also examined genetic polymorphisms in 13 genes related to MeHg metabolism for association with glioma risk; genetic associations were also studied in the UK Biobank cohort. Median toenail Hg in cases and controls, respectively, was 0.066 μg/g and 0.069 μg/g (interquartile range (IQR): 0.032–0.161 and 0.031–0.150 μg/g). Toenail Hg was not found to be significantly associated with glioma risk (Odds Ratio: 1.02; 95% Confidence Interval: 0.91, 1.14; p = 0.70 in analysis for ordinal trend with increasing quartile of toenail MeHg). No genetic variant was statistically significant in both of the studies; one variant, rs11859163 (*MMP2*) had a combined p-value of 0.02 though it was no longer significant after adjustment for multiple testing (Bonferroni corrected p = 1). This study does not support the hypothesis that exposure to MeHg plays a role in the development of glioma at levels of exposure found in this study population.

## Introduction

Gliomas are tumors arising from glial cells of the brain, representing approximately 80% of adult malignant brain tumors^1^. Etiology of glioma is poorly understood. However, several lines of evidence point to environmental risk factors. A higher incidence has been observed in Caucasians and in males regardless of race^2^. Incidence rates have also been found to vary geographically with rates being approximately two-fold higher in more developed compared to less developed regions^3^; geographic variation is also observed within the US^4^. Brain tumor mortality has been noted to be higher among immigrants to Canada^5^ and the US^6^ when compared to native-born populations, regardless of gender, suggesting geographical variation in the prevalence of causal agents or risk factors. However, to date, the only established environmental risk factor is ionizing radiation from medical and environmental exposures^7^. No study has examined the relationship between exposure to methylmercury (MeHg), an established neurotoxin, with glioma risk.

MeHg is a ubiquitous environmental contaminant associated with increased oxidative stress and inhibition of several important antioxidants^8^. Fish and shellfish comprise the major source of MeHg exposure in humans^9^. MeHg readily crosses the blood brain barrier (BBB) via L-type large neutral amino acid transporters (*LAT*)^10^. *SLC7A5* (*LAT1*) has been thought to play a role in cell proliferation and as a nutrient transport system at the BBB^11^, while *SLC7A8* (*LAT2*) plays a role in amino acid transport; both are suspected of transporting MeHg into different cells^12^. The ability of MeHg to cross the BBB makes the brain highly susceptible to the effects of MeHg-related neurotoxicity. The brain has a strong affinity for MeHg showing concentrations 3–6 times that found in the blood^13,14^. MeHg is highly neurotoxic and is known to accumulate in neural astrocytes, cells in the brain that can develop into gliomas^15,16^. Due to variability of MeHg content between and within^17^ species of fish, and changes in consumption over time, toenail mercury (Hg) concentration offers an etiologically relevant measure of dietary exposure and long-term (6–12 months) intake of MeHg^18–20^. Toenail Hg has been shown to approximate MeHg concentrations in the brain in rodent models as well as humans^21,22^; one study found Hg levels measured in toenails to be significantly correlated with MeHg levels measured in brain tissue taken from the occipital lobe^22^.

Epidemiologic studies on the relationship between MeHg consumption and cancer risk are lacking. *In vitro* evidence suggests that MeHg disrupts mitochondrial function and triggers overproduction of reactive oxygen species (ROS) which leads to cellular and DNA damage^23^. Glutathione (GSH) is an important intracellular antioxidant that reacts with ROS to limit free radical damaging effects in the cell, regulated by the selenoprotein glutathione peroxidase (GPx)^24^. MeHg limits the cellular uptake of the neurotransmitter glutamate, a precursor in GSH synthesis^15,25^. GSH prevents MeHg induced oxidative stress by binding to the molecule forming a conjugate (GS-HgCH_3_) that can be expelled in bile or urine^24,26^, increasing the glutathione disulfide (GSSG): GSH ratio, in turn reducing the antioxidant capacity in astrocytes and microglia^24^. Given the large body of research that provides evidence that oxidative stress induces carcinogenesis, and the ability for MeHg to readily cross the BBB via the *LAT* system, it is plausible that MeHg exposure may increase the risk for glioma onset^10,27^.

Single nucleotide polymorphisms (SNPs) in genes that regulate uptake, distribution, metabolism, and elimination of MeHg may influence an individual’s susceptibility to MeHg related neurotoxicity. Epidemiologic studies have suggested that the response to MeHg may be influenced by molecular variants in several genes that are involved in absorption, distribution, metabolism, and excretion^14^.

The aim of this study was to investigate the association between toenail Hg concentrations and genetic variants involved in the toxicokinetics of MeHg in relation to glioma risk in a US case-control study of glioma and the UK Biobank Cohort^28^.

## Subjects and Methods

### Study populations and toenail Hg measurement

#### Case-control study

Subjects were enrolled in a case-control study of glioma risk factors conducted at medical centers in the Southeastern US (‘GliomaSE’). A description of the study can be found in previous reports^29–32^. In brief, incident glioma cases were identified in neuro-oncology departments at participating academic medical centers in 6 US states (Alabama, Florida, Georgia, Kentucky, and Tennessee). Noncancer controls were identified via telephone white page listings, or were friends and nongenetically related family members of the cases. Germline DNA was collected for study of germline variation in relation to glioma risk and toenail clippings were collected for studies of trace metals. The University of South Florida Institutional Review Board approved the study.

#### Cohort study

The UK Biobank (UKB) cohort was utilized to further examine SNPs of interest in association with glioma risk. The UKB cohort in full consists of 502,619 participants, ages 40 to 69, who were recruited from 2006 to 2010^28^. Anonymized genotypes and clinical data were downloaded as part of an approved protocol (Application #16944). This study utilized 313,868 genetically unrelated Caucasians, with no history of cancer at baseline (other than non-melanoma skin). Prospective cohort analyses were based on a total of 322 incident glioma cases, identified from the National Health Service Central Registers through November 30, 2014 for England and Wales and December 31, 2014 for Scotland residents^28^.

### Toenail Hg measurement and analyses

For purposes of the present investigation, toenail samples were analyzed for Hg in a subset of 300 consecutively enrolled glioma cases and 300 controls matched to individual cases on age (within 5 years), sex, and US state of residence. Toenail Hg concentrations were determined using instrumental neutron activation analysis as previously described^33^. Conditional logistic regression was used to estimate odds ratios (OR) and 95% confidence intervals (CI) for the association between Hg levels in toenails and glioma risk.

### Analysis of genotype associations

A total of 402 variants spanning 13 candidate genes that regulate uptake, distribution, metabolism, or elimination of MeHg (Supplementary Table 1) [14] tiled on the Affymetrix ‘UKBiobank’ array were identified based on GRCh37 gene coordinates. (No SNPs in one of the candidate genes, *MT1A*, were tiled on the array.) SNPs with a minor allele frequency <0.05 or Hardy-Weinberg equilibrium p-value < 10^−5^ in controls were removed along with any SNPs or samples with a call rate <95%. This filtering procedure was applied separately to GliomaSE and the UKB cohort and all analyses were restricted to Caucasians. ORs between genetic variants and glioma risk were determined by logistic regression under an additive model. To control for multiple testing, Bonferroni correction was independently applied and p-values combining GliomaSE and UKB were calculated from unadjusted p-values using the Fisher method^34^. Gene-level associations were also calculated separately in GliomaSE and UKB using the sum of chi-squares, Bonferroni correction test and minimum P value test [35]. Results were considered statistically significant for p-value < 0.05.

### Ethical approval

All procedures performed in studies involving human participants were in accordance with the ethical standards of the institutional and/or national research committee and with the 1964 Helsinki declaration and its later amendments or comparable ethical standards. The “GliomaSE” case-control study was approved by the University of South Florida Institutional Review Board.

### Informed consent

Informed consent was obtained from all individual participants included in the study.

## Results

Descriptive information for the case-control study is shown in Table 1. The case-control population was comprised of glioma cases aged 18–81 years, and controls aged 20–85 years, with a median age of 54 years in both case and control groups. The population was predominantly male (61.7%) and Caucasian (~95%). Glioma diagnoses were comprised of a higher frequency of glioblastomas (64.7%) than lower grade (35.3%) subtypes consistent with population incidence patterns in glioma.

**Table 1:** Descriptive statistics of GliomaSE cohort.

Characteristic	Toenail Samples					Genotype Samples				
	Cases		Controls		p-value*	Cases		Controls		p-value*
	(n = 300)		(n = 300)			(n = 1547)		(n = 1015)		
Age					Matched					0.002
Median	55		55			55		56		
Range	(18–88)		(20–86)			(18–92)		(18–88)		
Sex, N (%)					Matched					0.001
Female	118	(60.7)	118	(60.7)		642	(41.5)	488	(48.1)	
Male	182	(39.3)	182	(39.3)		905	(58.5)	527	(51.9)	
Race, N (%)					0.29					—
Caucasian	286	(95.3)	280	(93.3)		1547	(100)	1015	(100)	
Non-Caucasian	14	(4.7)	20	(6.7)		0	(0)	0	(0)	
State of Residence, N (%)					Matched					<0.001
Florida	152	(50.7)	154	(51.3)		683	(44.1)	347	(34.2)	
Alabama	71	(23.7)	70	(23.3)		274	(17.7)	130	(12.8)	
Georgia	35	(11.7)	35	(11.7)		114	(7.4)	111	(10.9)	
Tennessee	26	(8.7)	27	(9.0)		221	(14.3)	264)	(26.0)	
Other	16	(5.2)	14	(4.7)		255	(14.5)	163	(16.1)	
Toenail Mercury (μg/g)					0.23					—
Median	0.066		0.069			—		—		
Range	(<0.01–0.92)		(<0.01–2.63)			—		—		
Histology, N (%)					—					—
Glioblastoma	194	(64.7)	—			941	(60.8)	—		
Non-GBM	106	(35.3)	—			606	(39.2)	—		

*t-test conducted for toenail mercury analysis and age, chi-square test conducted for race, sex and state of residence.

Toenail Hg concentration ranged from 0 µg/g to 2.633 µg/g, with no significant difference in Hg levels found comparing cases (median: 0.066 μg/g; IQR: 0.084–0.161 μg/g) and controls (median: 0.069 μg/g; IQR: 0.031–0.150 μg/g). Case-control results for Hg nail concentration and glioma risk are shown in Table 2. Using a referent group for Hg concentration of ≤0.030 µg/g, no significant association was observed between increasing toenail Hg concentration and glioma risk (OR = 1.02; 95% CI 0.91, 1.14; p = 0.70 for increasing quintile of toenail Hg). Furthermore, we observed no associations when stratifying analysis according to glioma subtype i.e., glioblastoma versus lower grade glioma (not shown). Finally, no associations were observed between nail Hg concentration and glioma patient survival (not shown).

**Table 2:** Association between toenail mercury concentration and risk of glioma.

Hg (μg/g)	No. Cases	No. Controls	Multivariate OR (95% CI)*	p-value
0.000–0.030	66	74	Ref	—
0.031–0.055	59	53	1.30 (0.79, 2.15)	0.31
0.056–0.084	49	43	1.31 (0.78, 2.19)	0.31
0.084–0.161	51	60	0.90 (0.54, 1.51)	0.68
>0.162	75	70	1.22 (0.76, 1.96)	0.42
trend			1.02 (0.91, 1.13)	0.76

*Conditional logistic regression model matched for age (5 year groups), state of residence (FL, AL, GA, TN, other), sex, and batch.

Of the initial 402 candidate SNPs, 220 SNPs passed QC filters in both GliomaSE and UKB. Odds ratios and 95% CIs for all 220 SNPs are shown in Supplemental Table 2. In the case-control study, 6 SNPs in 3 genes were significantly associated with glioma risk prior to adjustment for multiple testing (Table 3). Divergent effects were observed in *MT4* with a harmful association for rs11643815 (OR = 1.19; 95% CI 1.01, 1.40; p = 0.04) but a protective association for rs17285449 (OR = 0.81; 95% CI 0.66, 0.99; p = 0.04). Two deleterious associations were observed in *MMP2* for rs2576550 and rs34373154 (OR = 1.21; 95% CI 1.01, 1.44; p = 0.04 and OR = 1.22; 95% CI 1.02, 1.46; p = 0.03) and one SNP (rs17859821) in *MMP2* was associated with decreased risk (OR = 0.82; 95% CI 0.68, 0.97; p = 0.03). A decreased risk was observed with each additional copy of the minor allele in rs11624694 (*SLC7A8*; OR = 0.83; 95% CI 0.72, 0.97; p = 0.02). In the UKB cohort, 4 genetic variants in 3 genes were found to be statistically significantly associated with risk of developing glioma. Both rs12751325 and rs3748682 in *MTF1* were associated with a reduced risk (OR = 0.82; 95% CI 0.69, 0.98;  p = 0.03 and OR = 0.82; 95% CI 0.69, 0.98; p = 0.03). A protective association for each copy of the minor allele was also observed in rs17183863 (*SLC7A8*) and rs7186103 (*MT4*) (OR = 0.66; 95% CI 0.45, 0.96; p = 0.03 and OR = 0.77; 95% CI 0.60, 0.98; p = 0.04). No variants remained statistically significant in either study after adjusting for multiple testing. One variant in *MMP2*, rs11859163, had a statistically significant combined p-value of 0.02 before adjustment, with a protective association observed in both UKB and GliomaSE for each copy of the minor allele.

**Table 3:** Association between genetic variants and risk of glioma.

Gene	SNP	GliomaSE				UKB				Combined p*
		MAF	OR	(95% CI)	p-value	MAF	OR	(95% CI)	p-value	
MTF1	rs12751325	0.29	0.97	(0.86–1.10)	0.68	0.29	0.82	(0.69–0.98)	**0**.**03**	0.10
	rs3748682	0.29	0.98	(0.87–1.11)	0.75	0.29	0.82	(0.69–0.98)	**0**.**03**	0.11
SLC7A8	rs11624694	0.17	0.83	(0.72–0.97)	**0**.**02**	0.19	1.02	(0.84–1.24)	0.08	0.08
	rs17183863	0.06	0.95	(0.76–1.20)	0.66	0.06	0.66	(0.45–0.96)	**0**.**03**	0.10
MT4	rs11643815	0.14	1.19	(1.01–1.40)	**0**.**04**	0.14	1.09	(0.88–1.36)	0.44	0.09
	rs17285449	0.09	0.81	(0.66–0.99)	**0**.**04**	0.09	1.06	(0.81–1.38)	0.70	0.12
	rs7186103	0.14	1.03	(0.88–1.21)	0.69	0.14	0.77	(0.60–0.98)	**0**.**04**	0.12
MMP2	rs17859821	0.11	0.82	(0.68–0.97)	**0**.**02**	0.10	0.91	(0.69–1.19)	0.48	0.07
	rs2576550	0.12	1.21	(1.01–1.44)	**0**.**04**	0.12	1.13	(0.90–1.42)	0.30	0.07
	rs11859163	0.40	0.90	(0.80–1.01)	0.06	0.40	0.85	(0.73–1.00)	0.05	**0**.**02**
	rs34373154	0.12	1.22	(1.02–1.46)	**0**.**03**	0.12	1.12	(0.89–1.41)	0.32	0.05

*P-values combined from unadjusted p-values using Fisher’s method.

At a gene-level, only *MT4* in the GliomaSE cohort was significantly associated with glioma across all three tests (p = 0.04). No gene-level associations were statistically significant in UKB and no genes were significantly associated with toenail mercury levels.

## Discussion

This study found no association between toenail Hg and glioma risk. Multiple germline variants in pathways associated with MeHg metabolism were associated with glioma risk in either the case-control or cohort study; however, no SNP remained significant after adjustment for multiple testing. One variant in *MMP2* (rs11859163) had a statistically significant p-value of 0.02 when combining the two studies, with both studies suggesting a protective association; however, the result was nonsignificant after adjustment for multiple testing and nail mercury was unrelated to genotype for this SNP. *MT4* was the only gene significantly associated with glioma at a gene-level, with a significant SNP-level association observed in two of the twelve SNPs (16.7%) tested in this gene.

To the best of our knowledge, this is the first population study to examine MeHg as a risk factor for glioma and is unique in cancer studies by examining both a biomarker of exposure (toenail Hg) and polymorphic variation in MeHg metabolism^35^. Crespo-Lopez *et al*. examined the genotoxic effects of MeHg on glioma cell lines and found that even low levels of MeHg can cause disruptions to the cell cycle and cell proliferation^36^. One study following the outbreak of Minamata disease (MD), which was caused by Hg poisoning in 1953, showed no increased risk of dying from cancer overall in those with MD, while a decreased risk of death from stomach cancer and an increased risk of death from leukemia was observed, although the numbers in the study were small^37^. Janicki *et al*. found an increased concentration of Hg in hair samples from leukemia patients, specifically those with acute leukemia, compared to healthy controls^38^. Studies following the MeHg poisoning in the 1970s in Iraq showed similar results to studies performed after the MD outbreak and found evidence that in utero exposure impaired fetal development, such as mental and sensory impairments, paralysis and cerebral palsy^39^. In spite of such adverse effects in the CNS, the present study offers no support for a role of MeHg in the onset of glioma in adults.

With respect to generalizability, it is important to consider how mercury exposures in the present study compare with those in prior studies and with average population exposures. Toenail Hg concentrations were substantially lower in the present study population when compared to those reported in several other US-based^18,40–42^ studies, and studies outside the US^35,43–47^, several of which involved toenail measurements performed in the same laboratory as in the present study^35,42,45^. The primary source of MeHg exposure in humans is the dietary consumption of fish, with highest concentrations found in longer-lived, larger fish that bioaccumulate MeHg^8,9,48^. Relevant to this, subjects in the present study completed a questionnaire on diet patterns 5 years prior to glioma diagnosis/or a corresponding reference date in controls that included a question on frequency of consumption of dark meat fish (‘including salmon, mackerel, sardines, or swordfish’) (272 cases and 284 controls completed the questionnaire) and, similar to previous studies^40,43^, we observed modest though statistically significant correlation between reported servings of fish and toenail Hg overall (Pearson’s r = 0.25; p = 2.9e-9), and separately in cases (Pearson’s r = 0.31; p = 1.4e-7) and controls (Pearson’s r = 0.22; p = 0.0002). Fish consumption was lower than that reported in other study populations (only 2.2% reported >1 serving per week as compared to 27.8% among participants in two large US-based cohort studies^18^, explaining low MeHg exposure in the current study population. Therefore the present study offers a test of the hypothesis relating low to moderate levels of MeHg exposure to glioma risk.

Previous studies have identified multiple genetic variants associated with Hg in either hair or toenail samples, or other body tissues^14^. Among 202 controls with both toenail Hg and genotype data in the present study, two SNPs associated with body burden of mercury in previous studies (rs1050450 and rs1128503) likewise predicted toenail Hg in the present data; neither SNP was significantly associated with glioma risk (Supplemental Table 3). Several other variants in the 12 candidate genes were nominally associated with toenail Hg, none associated with glioma risk in either study (Supplemental Table 2**)**. Among the examined SNPs, toenail Hg was significantly associated with a single genetic variant (rs77878228 in *SLC7A5*) after correcting for multiple testing (beta = 0.27; p = 1.12e^−5^). To our knowledge, the variant has not been reported previously to affect body burden of MeHg^14^.

This study offers many strengths as well as some limitations. Incident glioma cases were enrolled rapidly upon diagnosis (generally within 1 month) and great toenails reflect dietary exposure 6–12 months in the past^49^ and thus preceding effects of illness and treatment on usual diet. Furthermore, reasonably high correlations have been reported for Hg in toenail samples collected 6 years apart^50^. For these reasons, toenail Hg in the present study provided a reasonably robust measure of long-term (pre-diagnostic, among the cases) exposure to MeHg among study subjects. Among glioma cases, no correlation was observed between toenail Hg and elapsed time from glioma diagnosis to toenail collection (Pearson’s r = 0.0002; p = 1). In the present study, we observed highly significant variation in toenail Hg in subjects stratifying by US states of residence (see Table 1); however, matching cases and controls on state of residency avoided potential confounding on regional differences in fish consumption. Associations between glioma risk and genetic variants in MeHg metabolism were based on a large case control study for a rare tumor and we attempted to validate associations in an independent cohort, with both studies using the identical ‘UK Biobank’ genotype array. Relationships of glioma risk with genetic variation in several additional genes potentially linked to mercury metabolism including *GPX1*, *GPX4* and *SEPP1*^14^ were considered in a previous report from the same cohorts (GliomaSE and UKB) [52]; one variant in *GPX1* (rs1050450) previously linked to mercury levels in body tissues^14^ was also found to predict nail mercury concentrations in the present study (Supplemental Table 3); however, neither this variant nor any other in these genes was significantly associated with glioma risk [52]. Among limitations, the sample size for toenail analyses was limited (300 cases and 300 controls) and we lacked power to evaluate associations according to other glioma risk factors, or independent of consumption of fish that contains potentially beneficial omega-3 fatty acids, and which is highly correlated with toenail Hg measures^51^. Finally, our study population based in the southeastern US reported relatively low intake of fish and had correspondingly limited exposure to MeHg; it is possible that only higher levels of MeHg than studied here have a demonstrable impact on glioma risk.

In summary, this study offers no support for a role of MeHg exposure in the development of glioma. Although there was limited overlap in associations of genetic variants with glioma risk in both the case-control study and UKBiobank cohort, some variants had suggestive findings and merit further investigation.

## Supplementary information

supplemental table 1, supplemental table 2, & supplemental table 3

## Data Availability

The datasets generated during and/or analyzed during the current study from the GliomaSE study are available from the corresponding author upon reasonable request.
